# Preoperative assessment of longitudinal extent in hilar cholangiocarcinoma using noninvasive enhanced MR radiomics: a multicenter study

**DOI:** 10.3389/fonc.2025.1632630

**Published:** 2025-09-05

**Authors:** Xin Quan, Xinqiao Huang, Jiong Liu, Xiang Yuan, Jian Shu

**Affiliations:** ^1^ Department of Radiology, The Affiliated Hospital of Southwest Medical University, Luzhou, Sichuan, China; ^2^ Department of Radiology, The Jiangsu Province Hospital of Chinese Medicine Chongqing Hospital (Chongqing Yongchuan Hospital of Chinese Medicine), Chongqing, China

**Keywords:** longitudinal extent, MRI, radiomics, Bismuth-Corlette classification, Hilar cholangiocarcinoma

## Abstract

**Objective:**

This study aims to develop a noninvasive radiomics model based on magnetic resonance imaging (MRI) for accurately predicting the longitudinal extent of hilar cholangiocarcinoma (HCCA), to assist in subsequent surgical decision making.

**Methods:**

This study retrospectively collected and analyzed data from patients with HCCA across three medical centers in China. Radiomics quantitative features were extracted from T2-weighted imaging (T2WI), diffusion-weighted imaging (DWI), and enhanced T1 high-resolution isotropic volume examination (e-THRIVE) sequences. L1 regularization was employed to select features, and three single-sequence radiomics models were developed to predict Bismuth type IV of HCCA. To improve the predictive accuracy for Bismuth type IV, the fusion model integrating the three single-sequence models was constructed. The performance of these models was evaluated comprehensively, and the optimal radiomics model for predicting longitudinal extent was identified.

**Results:**

A total of 154 patients with HCCA were included in the analysis. The radiomics models based on T2WI, DWI, and e-THRIVE sequences demonstrated predictive capabilities, with AUC values in the training set of 0.867, 0.923, and 0.872, respectively, and AUC values in the test set of 0.809, 0.823, and 0.808, respectively. The fusion model, which combined features from all three sequences, achieved superior predictive performance, with an AUC of 0.980 in the training set and 0.907 in the test set. This model demonstrated robust potential for predicting whether the HCCA was classified as Bismuth type IV.

**Conclusion:**

The multi-sequence MRI-based radiomics model can effectively predict Bismuth type IV of HCCA, assisting in clinical surgical decision-making, facilitating R0 resection to improve the prognosis of patients with HCCA.

## 1 Introduction

Cholangiocarcinoma is a rare but highly aggressive epithelial malignancy of the bile ducts and represents the most common malignancy of the biliary system (1). Over the past few decades, the global incidence of cholangiocarcinoma has steadily increased (2). Due to its asymptomatic nature in the early stages or the presence of non-specific symptoms, such as abdominal pain, weight loss, loss of appetite, pruritus, and jaundice, diagnosis is often delayed, resulting in a poor prognosis. Hilar cholangiocarcinoma (HCCA), originating at the bile duct confluence or the left and right hepatic ducts (3), is the most common subtype of cholangiocarcinoma, accounting for approximately 40-60% of all cases (4). Surgical resection remains the most effective treatment for HCCA (4). However, due to the complex anatomy of the hilar region and its proximity to vital structures, such as the arteries, portal vein, and liver parenchyma, HCCA presents significant challenges in terms of surgical approach, diagnosis, and prognosis (5). Achieving curative resection is particularly difficult, with only 30-50% of patients achieving R0 resection, and postoperative survival rates range between 20% and 40% (6). In patients with unresectable HCCA, palliative surgeries fail to extend postoperative survival (7). Consequently, accurate preoperative assessment of resectability is essential for optimizing patient outcomes.

Given the aggressive nature of HCCA, numerous studies have identified negative surgical margins (R0 resection) as the strongest predictor of long-term survival after surgery (8–10). Patients with positive margins have significantly worse survival outcomes compared to those achieving R0 resection (9, 11). Accurate assessment of the extent of HCCA invasion is crucial for surgical planning and achieving R0 resection, as this reduces the risk of recurrence and metastasis. In this context, preoperative assessment of the tumor’s longitudinal spread is essential for enabling curative resection (12). The Bismuth-Corlette classification, which focuses on the proximal degree of biliary tract involvement, is a highly intuitive tool for guiding surgical decisions (13). In previous studies, patients with Bismuth type I- III lesions were typically treated with bile duct resection or combined hemihepatectomy, depending on the direction of tumor extension (6, 14, 15). For Bismuth type IV lesions, extended hemihepatectomy or left/right trisectionectomy is commonly performed to ensure more complete removal of liver tissue (16–19), with concurrent total caudate lobectomy potentially achieving R0 resection (20). However, the expanded surgical extent significantly increases technical complexity, leading to prolonged operative time and increased blood loss, both of which substantially elevate the risk of postoperative complications (21). These may include liver failure, bile leaks, bilomas, intra-abdominal abscesses, and mortality (22, 23). A study indicated that Bismuth-Corlett type I-III (P = 0.009) was more likely to obtain R0 resection (24). Another research reported that ten-year OS was higher when comparing: Bismuth-Corlette I-III type tumor (7.7%) versus type IV tumors (2.7%) (25). Moreover, some cases of unresectable HCCA can be treated with liver transplantation following neoadjuvant therapy (13, 17).

Currently, MRI is widely recognized as the preferred imaging technique for staging HCCA in high-volume centers treating bile duct cancers (26–28). Compared to direct cholangiography, MRI is non-invasive and reduces the risk of tumor dissemination through surgical wounds. Additionally, MRI offers superior soft-tissue contrast compared to CT and ultrasound, making it crucial for evaluating bile duct involvement and aiding surgical planning. It is also more affordable than positron emission tomography (PET). Studies have shown that delayed periductal enhancement in dynamic contrast-enhanced MR scans indicates tumor invasion and improves the accuracy in distinguishing between resectable and unresectable tumors (29, 30). However, traditional MRI imaging methods, are reliant on the subjective interpretation of radiologists. On one side, HCCA is often accompanied by chronic inflammation or fibrosis of the surrounding bile duct walls, and the MRI signal characteristics are similar with those of tumor infiltration, making it difficult to distinguish them during visual assessment. On the other side, HCCA can infiltrate longitudinally along the submucosa of the bile duct (with a range of up to 2–3 cm above and below the main tumor mass). However, plain MRI or enhanced scans cannot clearly show such microscopic infiltration, and the extent can only be inferred indirectly from the length of bile duct stricture. This may lead to delayed judgment on “whether the tumor has invaded the key branches of the hepatic hilum” and affect the accuracy of typing. Besides, conventional MRI images assessment of radiologists lack quantitative analysis and fail to capture important quantitative features within tumors, such as cellular, physiological, and genetic heterogeneity (31). Radiomics can mine massive potential features from medical images, breaking through the limitations of visual assessment. This data enables quantitative analysis of lesion details, improving the objectivity and accuracy of diagnosis, typing, and prognostic evaluation (31, 32). Previous studies have used ultrasound radiomics to predict the longitudinal invasion (33), but no study has explored the use of MR Radiomics to predict the longitudinal invasion of HCCA.

The study aims to utilize MRI radiomics features derived from different sequences to accurately predict the longitudinal extent of HCCA. This will help determine the appropriate extent of surgical resection, guide the selection of the most suitable surgical approach, and ultimately improve patient prognosis.

## 2 Materials and methods

### 2.1 Patients characteristics

**Figure 1:**
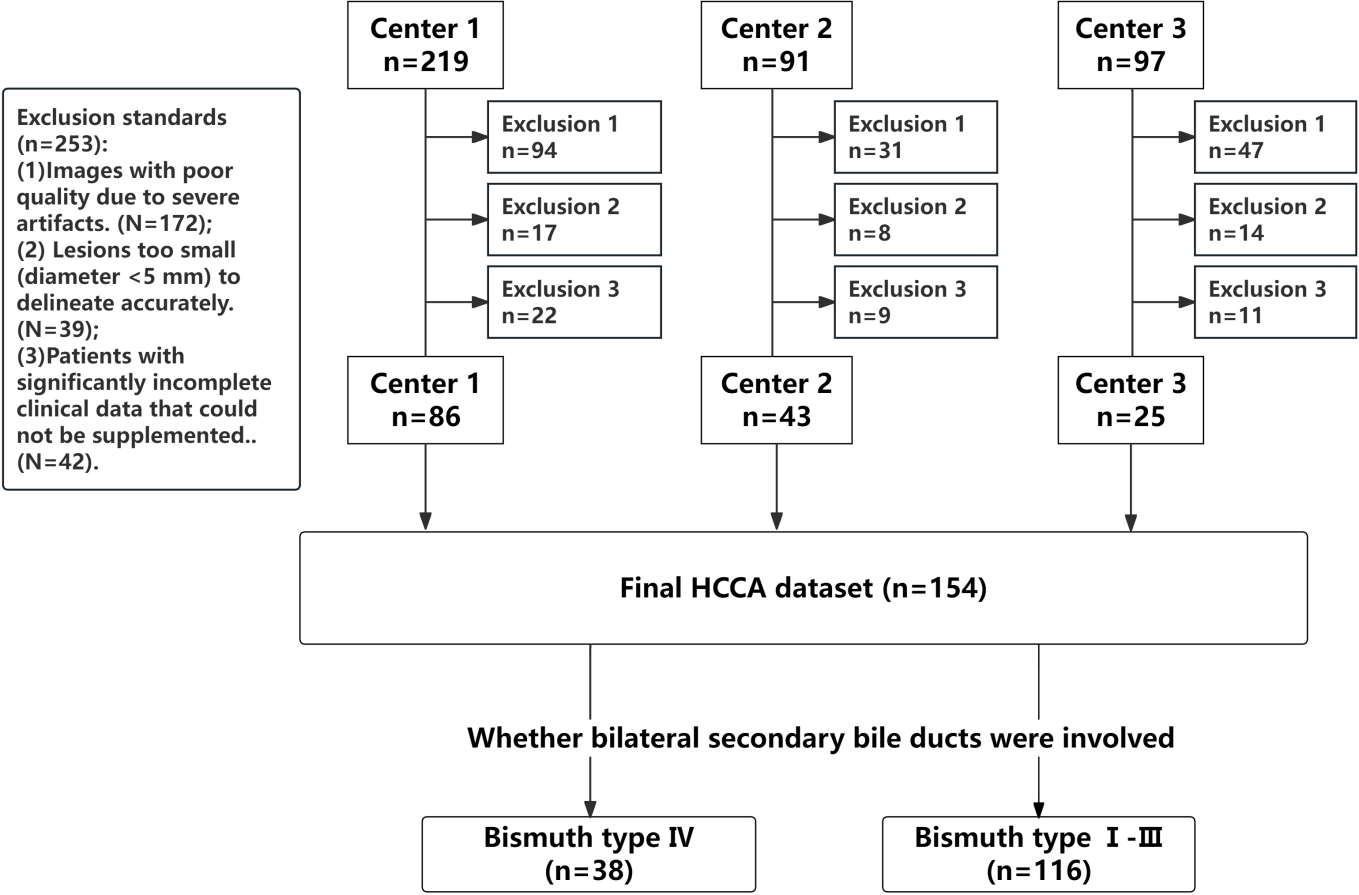
Flow chart of inclusion and exclusion.

This retrospective study was conducted in accordance with the ethical guidelines set by the Ethics Committee of our hospital (Approval Number: KY2024115). This research was supported by the National Natural Science Foundation of China (Project No. 82272077) and Science & Technology Department of Sichuan Province (Project No. 2024JDRC0045). Informed consent was waived due to the retrospective, multicenter nature of the study. The study included patients with confirmed HCCA who underwent surgical treatment and met the inclusion and exclusion criteria. The inclusion criteria included: (1) Patients who accepted surgical resection and pathologically diagnosed with HCCA; (2) Patients who underwent MRI examination within one month before surgery, with complete multi-parametric MR imaging data available. Patients were excluded for any of the following criteria: (1) Images with poor quality due to severe artifacts; (2) Lesions too small (diameter <5 mm) to delineate accurately; and (3) Patients with significantly incomplete clinical data that could not be supplemented. All pathological, clinical, and imaging data of the patients were collected. Patients were recruited from three medical centers, as summarized in **Figure 1**.

Bile duct invasion was classified for all patients using the Bismuth-Corlette classification system (19, 29), The specific types of classification are shown in **Appendix S1**. Bismuth type IV was divided into a group characterized by tumor involvement of bilateral secondary bile duct confluence or tumor invasion of hepatic hilum more than 2 cm, which was judged to be unresectable or required extended resection. Patients with Bismuth type I- III were classified into the other group.

This study incorporated a comprehensive set of clinical features, including sex, age, tumor location, Carbohydrate Antigen 19-9 (CA19-9), Aspartate Aminotransferase (AST), Alanine Aminotransferase (ALT), Total Bilirubin (TBIL), Direct Bilirubin (DBIL), Gamma-Glutamyl Transferase (GGT), presence of chronic hepatitis B, and history of Percutaneous Transhepatic Cholangial Drainage (PTCD).

### 2.2 MRI acquisition

Preoperative MRI examinations were performed on 1.5T or 3.0T superconducting whole-body scanners equipped with 16-channel abdominal coils. Standardized configurations across participating centers comprised: Center 1 using a 3.0T Philips Achieva scanner (Amsterdam, Netherlands), Center 2 utilizing 3.0T Siemens Avanto system (Erlangen, Germany), and Center 3 operating a 1.5T Siemens Prisma systems (Erlangen, Germany). Standardized pre-examination preparations and core acquisition parameters were maintained across all three centers. The scanning range extended from the top of the diaphragm to the umbilical level, covering the entire biliary system. Patients were instructed to fast and refrain from drinking for 4–8 hours prior to the examination. Before scanning, they were trained to maintain consistent breathing and perform end-expiratory breath-holds. For patients unable to hold their breath adequately, prospective respiratory gating techniques were employed to minimize motion artifacts caused by respiration.

The imaging sequences acquired included, but were not limited to, transverse T2-weighted imaging (T2WI), transverse diffusion-weighted imaging (DWI), and transverse enhanced T1 high-resolution isotropic volume examination (e-THRIVE). Sensitivity encoding (SENSE) technology was applied during image acquisition to reduce scan time while preserving image quality. For the e-THRIVE contrast-enhanced sequence of Center 1, gadobutrol was administered as the contrast agent at a dose of 0.2 mL/kg and an injection rate of 2/2.5 mL/s. Four-phase contrast-enhanced scans were performed at 20-30s, 60-70s, 120-130s, and 180-200s after contrast injection. *b* = 800/1000 s/mm^2^ images were used for DWI and Delayed-phase images were used for e-THRIVE. The parameters are shown in **Supplementary Table S1** in the Supplemental Material, and the specific parameters of enhanced scanning in each hospital are shown in **Supplementary Table S2**.

### 2.3 Radiomics analysis

The radiomics workflow in this study consisted of the following steps: lesion segmentation, feature extraction, feature selection, model construction, and model evaluation.

#### 2.3.1 Lesion segmentation

**Figure 2:**
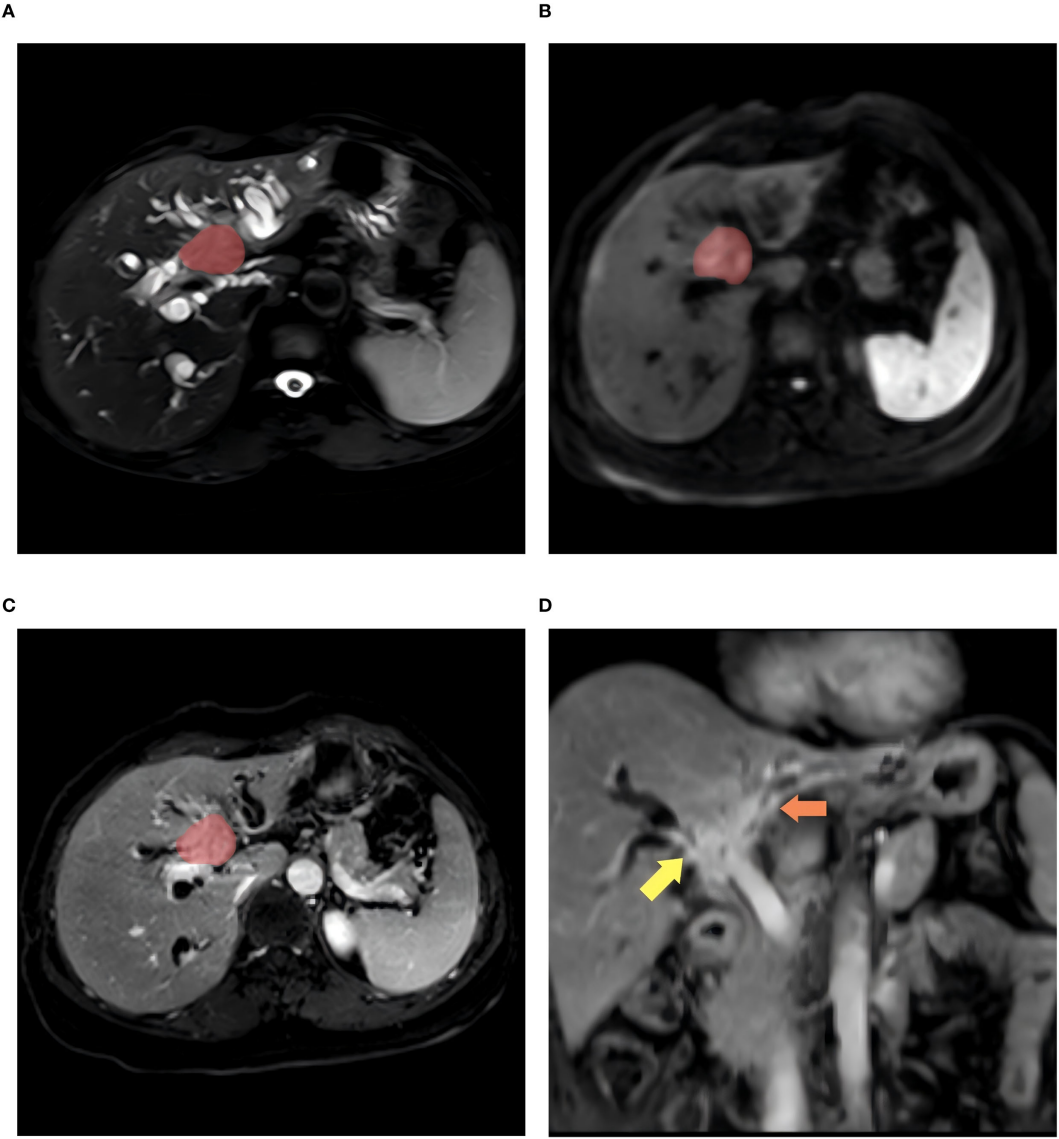
A 56-year-old woman with Bismush type IV HCCA. **(A–C)** shows the patient’s axial T2WI, DWI, and enhanced ROI contoured images, respectively, and **(D)** images show the affected areas at the bifurcation of the right hepatic duct and the left hepatic duct (indicated by the yellow arrows and the orange arrows).

All MR images were retrieved from the Picture Archiving and Communication Systems (PACS) and exported in DICOM format for all patients with HCCA, including the preoperative T2WI, DWI, and e-THRIVE sequences. The images were then imported into the Deepwise Multimodal Research Platform (version 2.2, https://keyan.deepwise.com, Beijing Deepwise & League of PHD Technology Co., Ltd, Beijing, China), hereafter referred to as the Deepwise Multimodal. HCCA lesions were segmented using an automatic segmentation model (34) previously published by our center, followed by calibration by two radiologists (Reader A and Reader B). For DWI images, segmentation was performed on the slice with a b-value of 800, while for e-THRIVE images, segmentation was conducted on delayed-phase images, as they provide high focal-liver contrast in the hepatobiliary phase (35). Regions of interest (ROIs) were delineated for all three MRI sequences for each patient, excluding obvious internal tumor structures such as visible blood vessels, necrotic or cystic areas, hemorrhagic regions, and adjacent dilated bile ducts. The ROI was drawn to keep an approximate distance of 1e2 mm from the tumor margin with reference to the MRI images. The manual correction of tumor boundaries was performed through integrated analysis of multi-sequence MRI features, specifically combining ductal structural alterations (dilation/stenosis) on T2-SPAIR, hyperintense tumor foci on DWI indicating restricted diffusion, and ductal enhancement patterns on e-THRIVE. Definitive indicators of bile duct invasion included ductal discontinuity or obstruction, non-visualization or irregular stenosis of distal segmental branches, asymmetric pre-stenotic dilation, intraductal filling defects, and enhancing irregular wall thickening (>3 mm) on dynamic contrast-enhanced phases, while excluding necrotic regions, hemorrhagic foci, and non-restricted adjacent dilated ducts (19). An example of HCCA in a patient with Bismuth type IV is shown in **Figure 2**.

#### 2.3.2 Feature extraction

Radiomics feature extraction in this study was also performed using the Deepwise Multimodal Research Platform (https://keyan.deepwise.com). Features were extracted separately for each imaging sequence—T2WI, DWI, and e-THRIVE—resulting in a total of 2158 features per sequence. These features were categorized into the following groups: First Order, Shape, Gray Level Co-occurrence Matrix (GLCM), Gray Level Size Zone Matrix (GLSZM), Gray Level Run Length Matrix (GLRLM), Gray Level Dependence Matrix (GLDM), and Neighboring Gray Tone Difference Matrix (NGTDM). Multiple filter transformations were applied to enhance texture feature extraction, including: Wavelet, Square, SquareRoot, Logarithm, Exponential, Gradient, Laplacian of Gaussian (LoG), LBP-2D, and LBP-3D transforms. For LoG filtering, the parameter for the kernel size of the LoG transformation is set to “1, 2, 3, 4, 5”. All images underwent intensity normalization and isotropic resampling to a uniform voxel size of 1× 1× 1 mm^3^. During radiomics feature extraction, the bin width parameter was fixed at 25 to standardize gray-level discretization.

To ensure the stability and reproducibility of the ROI segmentation used in this study, consistency testing was conducted on the MRI images of 20 randomly selected HCCA patients across the three sequences. Features with intraclass correlation coefficients (ICCs) ≥ 0.8 were retained, indicating good reliability, while those with lower ICC values were excluded from further analysis.

#### 2.3.3 Feature selection

Since feature dimension reduction may generate new, harder-to-interpret features, this method was not used in the study. Features with more than 10% missing values were excluded, while features with missing values ≤10% were filled with the average of the remaining values. The remaining radiomics features were standardized using the Z-score method to reduce dimensional differences between features. Given that the number of extracted features far exceeded the sample size, the machine learning process could become excessively slow and prone to overfitting. Therefore, it was necessary to perform stringent selection of high-dimensional and redundant features. Initially, correlation analysis was conducted among all features, and one feature from each pair with a linear correlation coefficient greater than 0.9 was removed. Subsequently, L1 regularization was applied for feature selection, and 15–20 features were retained for each imaging sequence based on the total number of cases.

#### 2.3.4 Model construction

Radiomics models were constructed for each imaging sequence using the selected features. To address limited sample size and class imbalance, a five-fold cross-validation approach was employed with strict preservation of the original data distribution in each fold. This enabled robust hyperparameter optimization based on validation performance. Three linear machine learning models—Logistic Regression (LR), Support Vector Machine (SVM), and Linear SVC—were trained, with the best-performing model on the test set selected as the final output.

The training and validation scores for the T2WI, DWI, and e-THRIVE sequence models were compiled into an Excel file and uploaded to the platform for model fusion. The fusion model allowed the use of different classifiers, including LR, SVM, Linear SVC, Decision Tree (DT), and Random Forest (RF). Five-fold cross-validation was also applied during the training of the fusion model. The final model was selected based on the highest AUC value achieved on the test set.

#### 2.3.5 Model analysis and evaluation

Receiver operating characteristic (ROC) curves were plotted for each model, and the area under the curve (AUC), accuracy, sensitivity, specificity, positive predictive value (PPV), and negative predictive value (NPV) were calculated to quantify the predictive performance of each model in the training, validation, and test cohorts. A model was considered strong if the AUC was ≥ 0.9, moderate if AUC was between 0.7 and 0.9, and low if AUC was between 0.5 and 0.7. An AUC value < 0.5 indicated no predictive ability. To further evaluate the performance of the optimal predictive model, additional analyses were conducted for both the training and test groups. These included precision-recall (PR) curves, decision curves, rad score distribution plots, and waterfall plots. In the PR curve, better performance is indicated by a curve closer to the upper right corner (both Precision and Recall near 1). In the decision curve, a higher curve position and a wider threshold coverage range signify greater clinical value.

### 2.4 Statistic analysis

SPSS software (IBM version 27.0) was used to analyze the clinical information and laboratory test results of HCCA patients. For continuous variables, the independent sample t-test or Wilcoxon rank-sum test was applied. Differences in categorical variables between groups were compared using the χ^2^ test. All statistical tests were two-sided, and P values < 0.05 were considered statistically significant.

## 3 Results

### 3.1 Patient characteristics and univariate analysis

**Table 1** summarizes the clinical characteristics of the patients included in this study (n = 154). The cohort consisted of 96 males (62%) and 58 females (38%), with ages ranging from 39 to 85 years and a mean age of 63 years. All tumors were confirmed as adenocarcinomas. Based on the tumor’s involvement of the confluence of bilateral secondary bile ducts or extension >2 cm beyond the hepatic hilum, patients were categorized into a Bismuth type IV (n = 38) and a Bismuth type I-III (n=116). Univariate analysis of clinical characteristics and laboratory tests showed no statistically significant differences between the groups (**Table 1**).

**Table 1:** Clinical and imaging characteristic analysis in patients with HCCA.

Factors	n	Bismuth-Corlette classification		P
		Bismuth type I-III (n=116)	Bismuth type IV (n=38)	
Age		63 (39-85)	61 (44-76)	0.106
Sex				0.613
male	96	71	25	
female	58	45	13	
CA19-9		556.479 ± 1132.253	339.93 ± 339.698	0.414
ALT		143.819 ± 131.54	198.803 ± 220.486	0.171
AST		116.748 ± 91.694	157.487 ± 173.25	0.254
TBIL		150.257 ± 115.785	170.408 ± 100.231	0.339
DBIL		112.015 ± 90.188	128.697 ± 74.240	0.304
GGT		662.543 ± 612.904	642.594 ± 621.629	0.864
CHB				0.195
Yes	11	6	5	
No	143	110	33	
PTCD				0.604
Yes	54	42	12	
No	100	74	26	

CA19-9, Carbohydrate Antigen 19-9; AST, Aspartate Aminotransferase; ALT, Alanine Aminotransferase; TBIL, Total Bilirubin; DBIL, Direct Bilirubin; GGT, Gamma-Glutamyl Transferase; CHB, chronic hepatitis B; PTCD, history of Percutaneous Transhepatic Cholangial Drainage.

Age is expressed as mean (min-max), and the remaining continuous variables are expressed as mean ± SD.

### 3.2 Inter-observer and intra-observer consistency in ROI delineation

Through intra-observer and inter-observer consistency analysis, 1,548 features with ICCs ≥ 0.8 were retained from the T2WI sequence, 1,636 from the DWI sequence, and 1,728 from the e-THRIVE sequence. These reliable features, demonstrating good consistency, were subsequently included in the feature selection and model construction processes.

### 3.3 Single sequence model construction and performance

**Figure 3:**
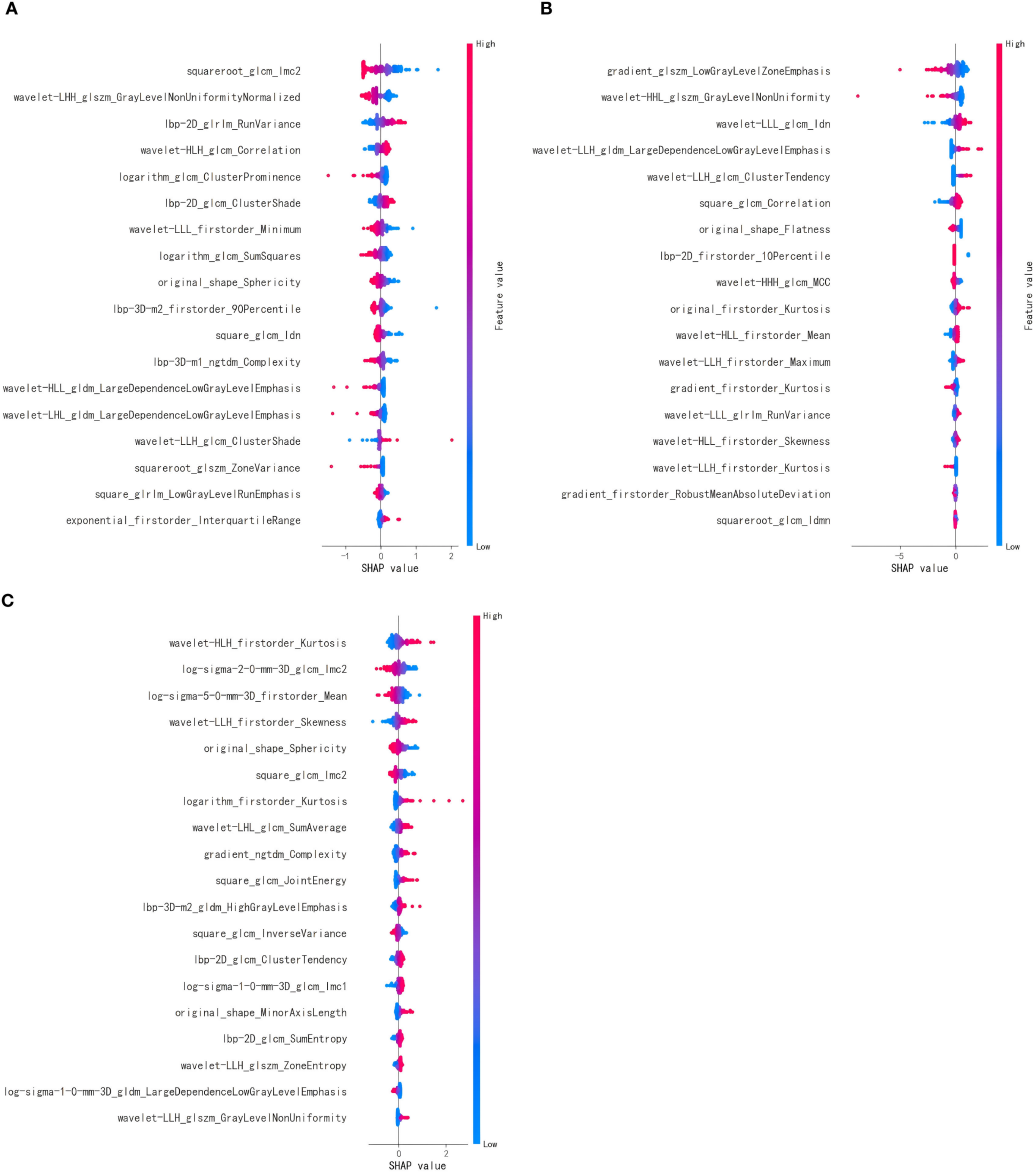
**(A–C)** show the SHAP diagram of optimal features of T2WI, DWI, and e-THRIVE, respectively. Each point represents a feature’s impact on predictions. Color gradient (red=high, blue=low) indicates feature value magnitude. Features are sorted by mean |SHAP| (descending). Horizontal axis: SHAP value quantifies directional influence- positive values increase the probability of predicting Bismuth type IV, while negative values decrease it.

**Figure 4:**
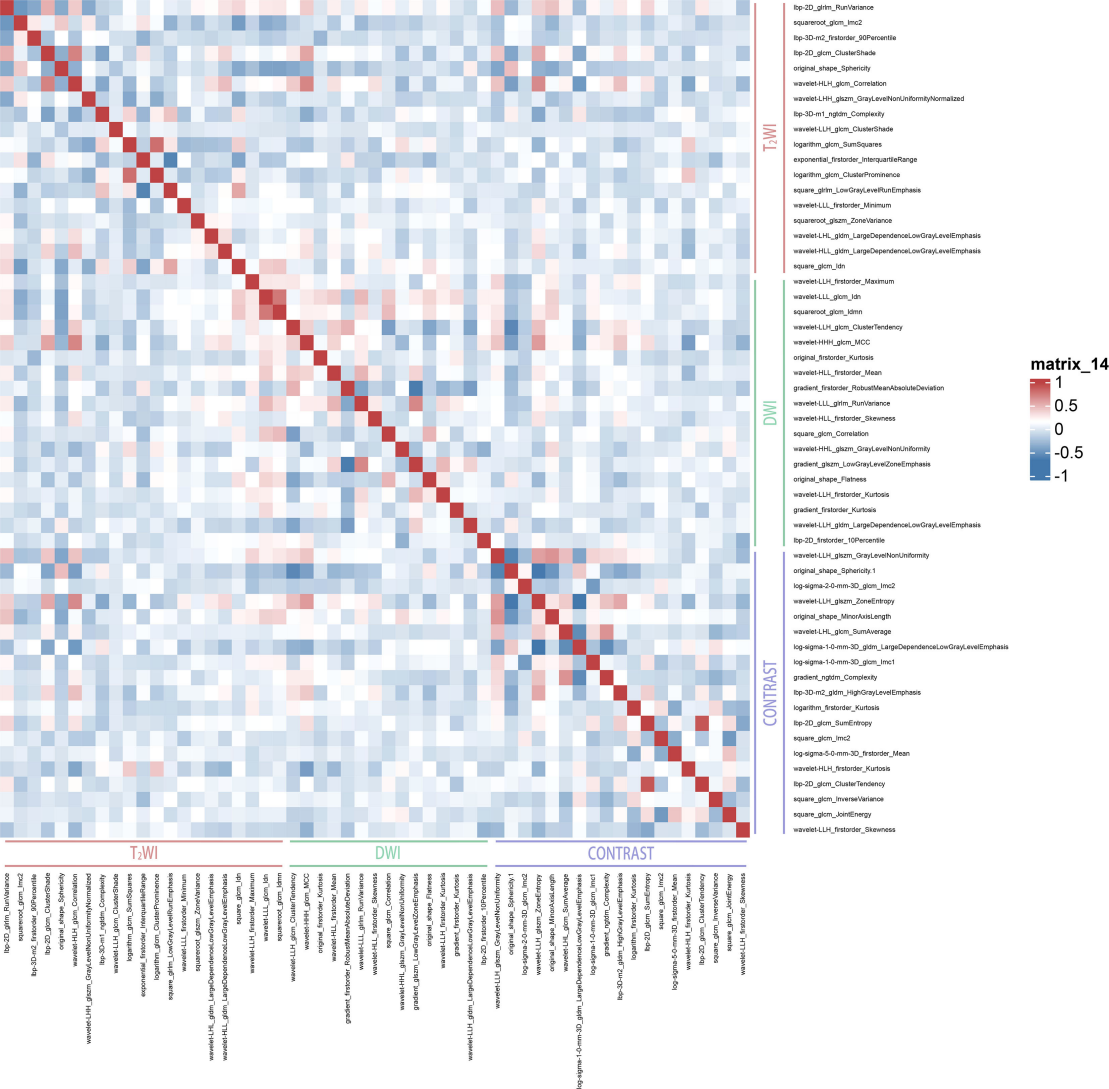
The heatmap of the selected features of roptimal features.

**Figure 5:**
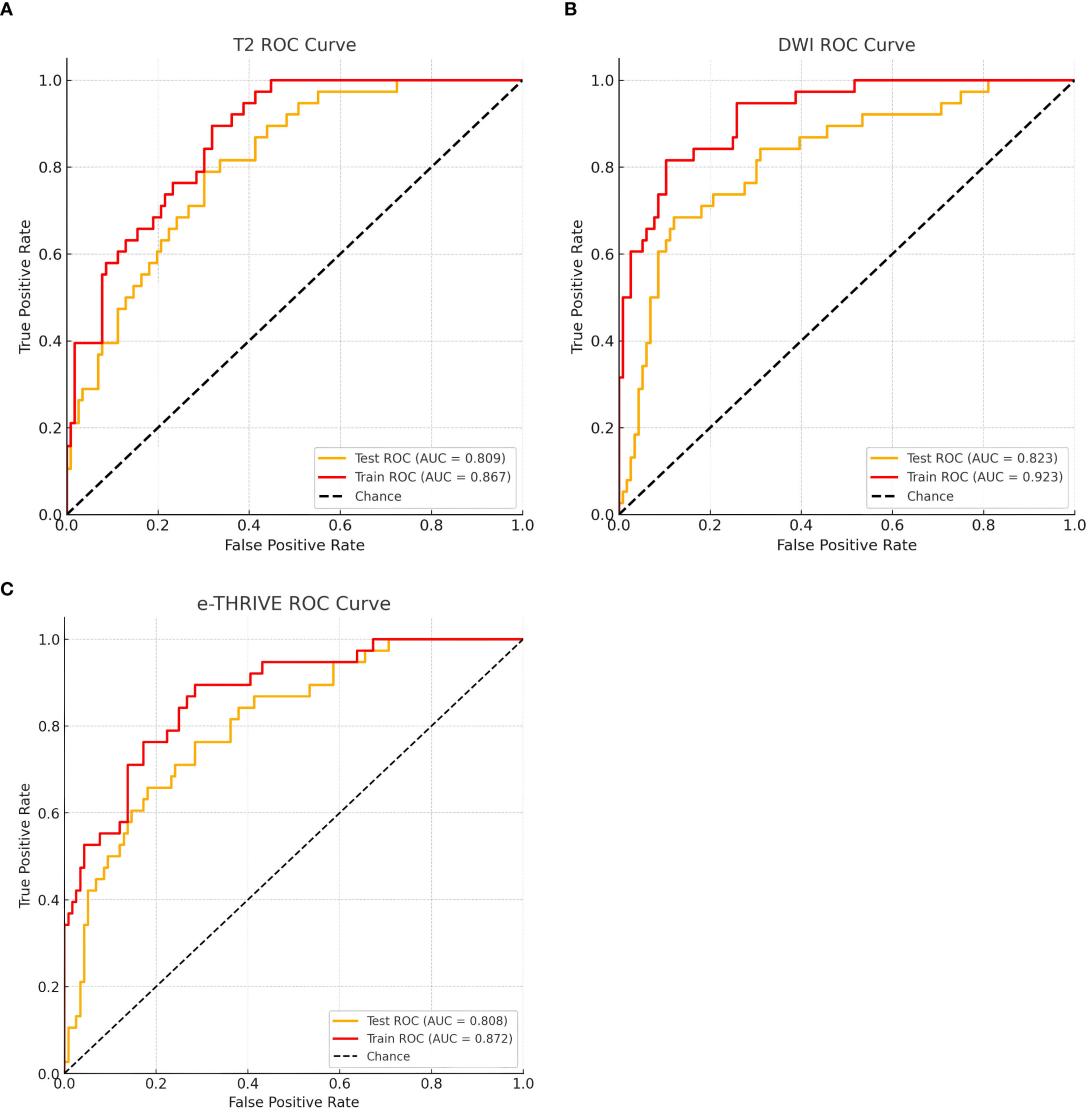
The ROC curve of radiomics model for predicting Bismuth type IV of HCCA. **(A–C)** show the T2WI, DWI, and e-THRIVE sequence models, respectively.

After feature selection, 18 optimal features were retained from the T2WI and DWI sequences, and 19 features were retained from the e-THRIVE sequence. The corresponding SHAP diagram is shown in **Figure 3**, SHAP analysis revealed that for the T2WI sequence, squareroot_glcm_lmc2, wavelet-LHH_glszm_GrayLevelNonUniformityNormalized, and lbp-2D_glrlm_RunVariance were the most critical features for predicting Bismuth type IV (**Figure 3A**). Among these, squareroot_glcm_lmc2 emerged as the most influential factor, reflecting local texture homogeneity and demonstrating a negative correlation with Bismuth type IV. In the DWI sequence, gradient_glszm_LowGrayLevelZoneEmphasis, wavelet-HHL_glszm_GrayLevelNonUniformity, and wavelet-LLL_glcm_Idn constituted the core predictive features (**Figure 3B**), with the dominant factor gradient_glszm_LowGrayLevelZoneEmphasis characterizing the distribution patterns of hypointense areas, also showing a negative correlation with Bismuth type IV. For the e-THRIVE sequence, predictive efficacy centered on wavelet-HLH_firstorder_Kurtosis, log-sigma-2-0-mm-3D_glcm_Imc2, and log-sigma-5-0-mm-3D_firstorder_Mean (**Figure 3C**), where the primary feature wavelet-HLH_firstorder_Kurtosis quantified extreme outlier distributions in delayed-phase enhancement intensity, exhibiting a positive correlation with Bismuth type IV. And the heatmap of the selected features is shown in **Figure 4**. Radiomics models were then constructed for each sequence, with ROC curves displayed in **Figure 5**. All three models demonstrated strong predictive performance for longitudinal extent in HCCA, with test set AUC values exceeding 0.8. The training set AUC values were 0.867, 0.923, and 0.872 for the T2WI, DWI, and e-THRIVE models, respectively, while the test set AUC values were 0.809, 0.823, and 0.808, respectively.

### 3.4 Model fusion and performance

**Figure 6:**
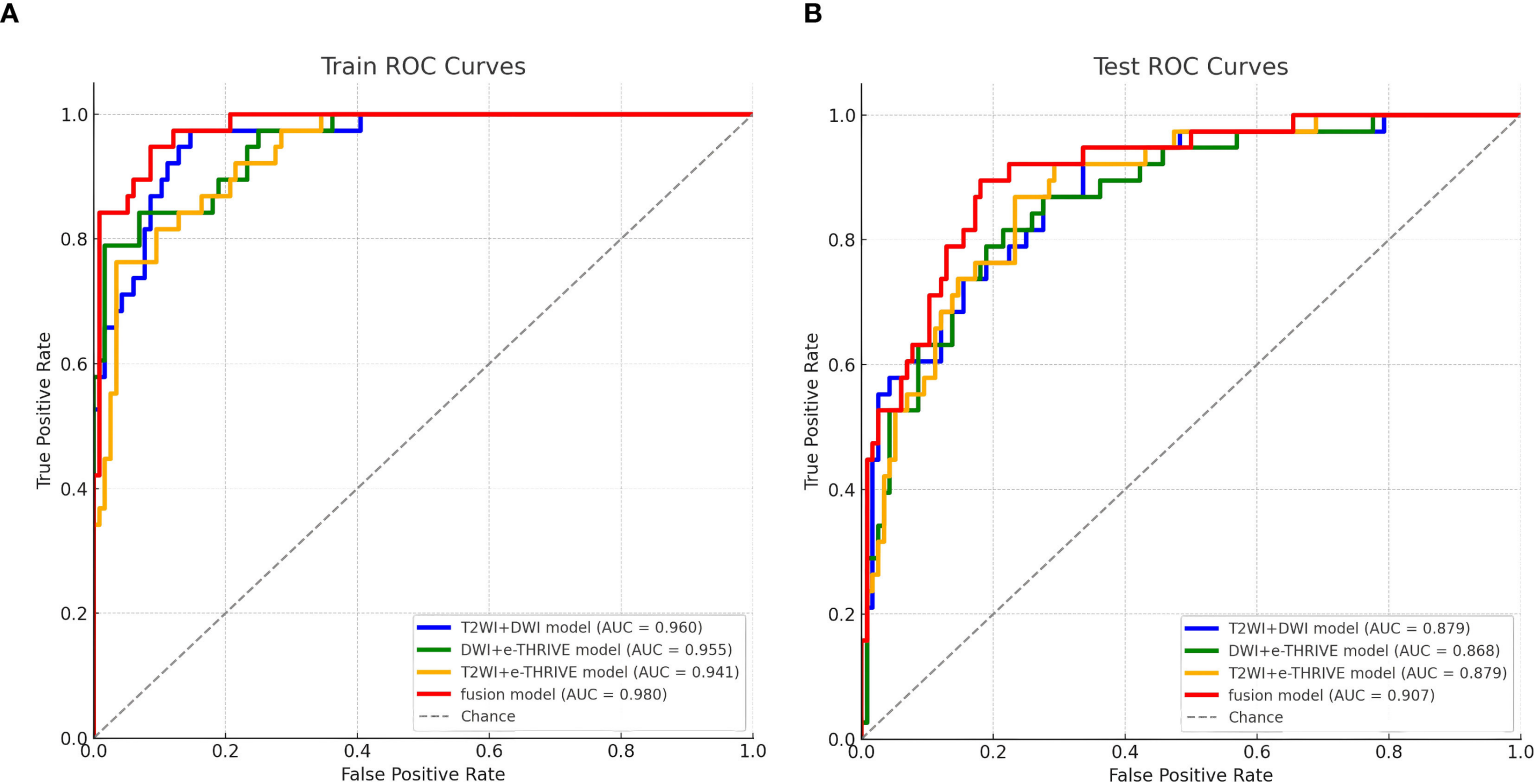
Fusion model ROC curve for predicting Bismuth type IV of HCCA. **(A, B)** show the training set and test set of the fusion model, respectively.

**Figure 7:**
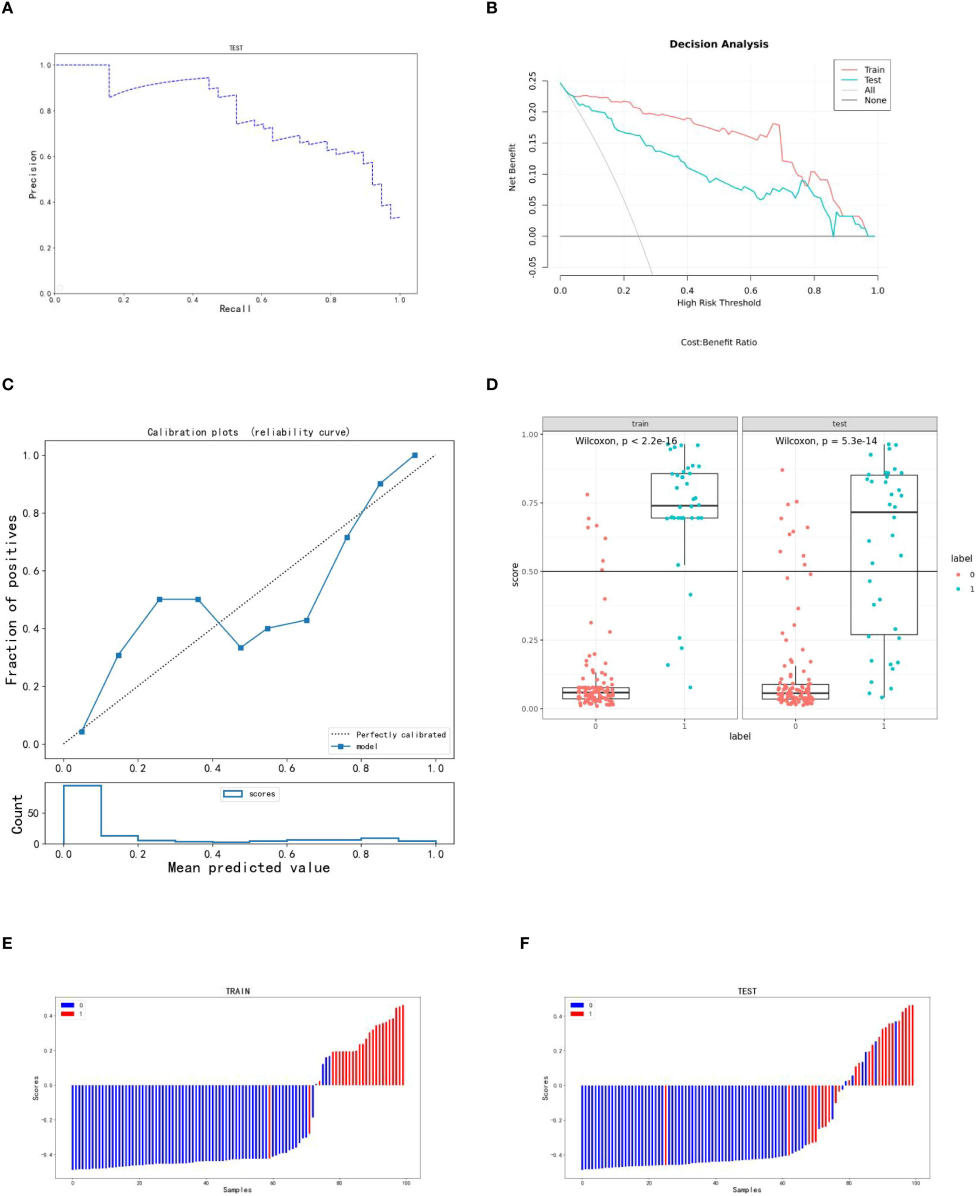
Indicators for predicting three sequence fusion models in patients with HCCA. **(A–C)** are the PR curve, decision curve and calibration curve of the fusion model respectively; **(D)** is the rad score diagram of the fusion model; **(E, F)** are the waterfall diagram of the training set and verification set of the fusion model respectively.

Using the scores from the three single-sequence models, a fusion model was constructed by integrating the T2WI, DWI, and e-THRIVE sequences. The fusion model demonstrated the best predictive performance, employing the SVM method, with a test set AUC value was 0.907(95% CI: 0.855-0.960). This model exhibited excellent predictive accuracy, with an accuracy of 0.834, sensitivity of 0.711, and specificity of 0.879. Additionally, fusion models combining any two sequences showed improved predictive performance compared to single-sequence models. Detailed evaluation metrics for these models are summarized in **Table 2**, and the corresponding ROC curves are shown in **Figure 6**. The PR curve (**Figure 7A**) demonstrated the model’s strong predictive ability for HCCA classified as Bismuth type IV. The decision curve (**Figure 7B**) confirmed the clinical value of the fusion model, showing that its net benefit in predicting Bismuth type IV was higher than the assumption that patients would develop Bismuth type I- III. The calibration curve (**Figure 7C**) demonstrated good agreement between the model-predicted probabilities and actual outcomes. Additionally, rad score distribution (**Figure 7D**) and waterfall plots (**Figures 7E, F**) visually supported the fusion model’s predictive ability for Bismuth type IV. DeLong’s test revealed no statistically significant differences among the models.

**Table 2:** Results of model evaluation indexes for predicting Bismuth type IV in HCCA patients.

Models	Methods	Data set	AUC 95% CIs		ACC	Sen	Spe	PPV	NPV
T2WI Model	LR	Train	0.867	[0.809-0.924]	0.701	0.921	0.629	0.449	0.961
		Test	0.809	[0.736-0.881]	0.649	0.816	0.595	0.397	0.908
DWI Model	LinearSVC	Train	0.923	[0.878-0.968]	0.864	0.632	0.940	0.774	0.886
		Test	0.823	[0.744-0.901]	0.838	0.605	0.914	0.697	0.876
e-THRIVE Model	LR	Train	0.872	[0.809-0.935]	0.766	0.895	0.724	0.515	0.954
		Test	0.808	[0.732-0.885]	0.643	0.816	0.586	0.392	0.907
T2WI+ DWI Model	LR	Train	0.960	[0.931-0.989]	0.890	0.737	0.940	0.800	0.916
		Test	0.879	[0.817-0.941]	0.812	0.605	0.880	0.622	0.872
DWI+e-THRIVEModel	LR	Train	0.955	[0.922-0.984]]	0.903	0.790	0.940	0.811	0.932
		Test	0.868	[0.802-0.933]	0.812	0.632	0.871	0.615	0.878
T2WI+e-THRIVE Model	SVM	Train	0.941	[0.904-0.977]	0.834	0.868	0.823	0.623	0.951
		Test	0.879	[0.820-0.938]	0.812	0.763	0.828	0.592	0.914
Three Sequences Fusion Model	SVM	Train	0.980	[0.963-0.998]	0.916	0.895	0.922	0.791	0.964
		Test	0.907	[0.855-0.960]	0.834	0.711	0.879	0.659	0.903

AUC, Area under the curve; AUC 95% CIs, 95% confidence interval of AUC; ACC, Accuracy; Sen, Sensitivity; Spe, Specificity; PPV, Positive predictive value; NPV, Negative predictive value. The result retains three decimal places.

## 4 Discussion

HCCA is a highly malignant tumor with complex anatomical structures and significant surgical challenges. Currently, precise diagnosis, staging, treatment, and prognostic evaluation for HCCA are inadequate. Surgical resection is still the only effective treatment. However, patients with positive surgical margins who do not achieve R0 resection generally have a poor prognosis, prone to recurrence and reduced overall survival (12). Enhancing preoperative assessment of longitudinal extent and Bismuth-Corlette classification in HCCA is crucial for optimizing clinical treatment strategies and improving patient survival.

In this study, we first selected 18 radiomic features from the T2WI and DWI sequences and 19 features from the e-THRIVE sequence through feature selection. Single-sequence models were constructed using three machine learning algorithms, and the best-performing models were identified. These optimal prediction scores from the single-sequence models were then integrated into a fusion model, which was constructed using five machine learning algorithms. The modeling process was repeated 100 times to ensure reliability and satisfactory predictive performance. Among the single-sequence models, the test set AUC values for the T2WI, DWI, and e-THRIVE models were 0.809, 0.823, and 0.808, respectively. The inverse association of gradient_glszm_LowGrayLevelZoneEmphasis with Bismuth type IV on DWI-SHAP plots (**Figure 3B**) reveals a critical pattern: diminished feature values indicate confluent diffusion-restricted areas, corresponding to high tumor cell density, intracellular water retention, increased necrotic and viscous components in the tumor, and narrowed extracellular space. This mirrors the longitudinal invasive growth characteristic of Bismuth type IV HCCA along bile ducts. The superior performance of the DWI model may be attributed to the fact that the DWI sequence takes advantage of the diffusion characteristics of water molecules in tumor tissues. In tissues rich in tumor cells, water diffusion is restricted, resulting in higher signal intensity on DWI and greater contrast, which helps differentiate abnormal from normal tissues (36, 37). In the two-sequence fusion models, combining DWI with T2WI or e-THRIVE consistently improved predictive performance. This may be due to e-THRIVE providing detailed information on capillary permeability, enhancing diagnostic accuracy, while the high contrast of DWI compensates for the relatively lower signal contrast of the other sequences. The fusion model combining T2WI, DWI, and e-THRIVE sequences demonstrated the highest predictive ability for longitudinal extent in HCCA, achieving an AUC of 0.907 (95% CI: 0.855-0.960). These results suggest that MR radiomics holds significant promise for preoperative assessment of longitudinal extent in HCCA, helping clinicians select the most appropriate treatment strategies and reduce recurrence and improve survival.

To our knowledge, this study is the first to utilize MR radiomics to predict bilateral secondary bile duct involvement in HCCA, integrating features from T2WI, DWI, and e-THRIVE sequences into a multimodal fusion model. Despite the rarity of HCCA and the retrospective nature of the study, we have made considerable efforts to explore and validate this novel diagnostic tool, representing a key innovation. Enhanced MR imaging provides valuable insights into bile duct involvement, while radiomics offers a non-invasive, comprehensive method to evaluate tumors and their microenvironment, capturing subtle variations missed by traditional imaging. This approach has great potential for improving diagnostic and prognostic accuracy (38–40). Several studies have explored radiomics in cholangiocarcinoma, such as using a radiomics nomogram to predict early recurrence of intrahepatic cholangiocarcinoma after hepatectomy (41), predicting tumor differentiation and lymph node metastasis in extrahepatic cholangiocarcinoma (ECCA) (42), and analyzing protein expression to guide treatment decisions (43). Recently, radiomics has also been used to predict microvascular invasion in HCCA (39) and lymph node metastasis in intrahepatic cholangiocarcinoma (44). Previous studies have evaluated the longitudinal extent of bile duct cancer using various imaging techniques. Okumoto et al (45). assessed four-channel multi-slice CT images in 18 patients, with correct diagnosis in 77.8% (14/18). Ryoo et al (46). used multi-row spiral CT, including MPR and MinIP images, to evaluate the longitudinal extent of bile duct cancer. The AUC for predicting longitudinal extent was 0.938 and 0.923 for MDCT MPR and MinIP images, respectively, and 0.839 and 0.836 for transverse MDCT MRC images in 27 patients. Hee et al (47).evaluated bilateral secondary bile duct confluence in 27 patients, with an overall accuracy of 90.7% using MRI combined with MRCP, and 85.1% using MDCT with direct cholangiography. Another study assessed the longitudinal extent in 15 patients using visual evaluation, with delayed-phase enhancement and MRCP images achieving accuracies of 93.3% and 80% (30), respectively. Hikaru et al (9) assessed bile duct longitudinal invasion using multi-row CT, ERCP, intraductal ultrasound, and biopsy, with 83.6% accuracy in 61 patients. Compared to prior studies, our research demonstrates significant advancements through a multicenter cohort of 154 patients—overcoming sample size limitations while enhancing result reliability. We implemented five-fold cross-validation that preserved natural class imbalance (Bismuth type IV vs. Type I- III), mirroring real-world incidence rates and strengthening clinical applicability. Critically, whereas traditional imaging assessments remain operator-dependent, our noninvasive radiomics model objectively quantified and integrated features from T2WI, DWI, and e-THRIVE sequences, breaking through the limitations of visual assessment and achieving superior predictive performance (test set AUC: 0.907, accuracy: 83.4%). The application of SHAP analysis further elucidated feature contributions, enhancing interpretability for clinical translation.

This study has several limitations. First, due to the low incidence of HCCA and the presence of incomplete medical records, the sample size was relatively small. Second, the dataset from some medical institutions is limited and still expanding. Future research should aim to supplement this data and perform external validation. Third, the retrospective design and the limited number of patients undergoing surgery with gold-standard pathological diagnosis introduce potential selection bias. Fourth, the lack of significant predictive clinical features may be attributed to the small sample size, limiting the representativeness of selected clinical factors. Fifth, the Bismuth-Corlette classification does not account for vascular involvement or lobar atrophy, which we hope to address in future studies to improve prediction accuracy for vascular and liver involvement.

## 5 Conclusion

The sequence fusion radiomics model based on MRI images demonstrates strong predictive ability for longitudinal extent in HCCA patients, providing valuable support for clinical decision-making. This model can help determine whether patients require extended surgical resection, ultimately improving medical efficiency, reducing costs, and significantly enhancing the prognosis of HCCA patients.

## Data Availability

The data from our institution are currently being used in ongoing follow-up studies and are not publicly available. Additional data were obtained from collaborating medical institutions under data-sharing agreements and are restricted to internal research use only. Requests to access these datasets should be directed to 552185250@qq.com.
