# Supplementary material for: Preoperative assessment of longitudinal extent in hilar cholangiocarcinoma using noninvasive enhanced MR radiomics: a multicenter study
Source: Front Oncol. 2025 Sep 5;15:1632630. doi: 10.3389/fonc.2025.1632630 (PMC12446029; doi:10.3389/fonc.2025.1632630)
Supplement: Supplementary file 1 [file DataSheet1.docx]

### Supplemental Material

### Appendix S1

### Bismuth-Corlette classification system

**Type I:** Tumor localized to the common hepatic duct, without involvement of the hepatic duct confluence (biliary bifurcation).

**Type II:** Tumor extends to and directly invades the hepatic duct confluence, but spares the proximal right and left hepatic ducts.

**Type IIIa/IIIb:** Tumor involves the hepatic duct confluence with proximal extension:

**IIIa:** Infiltration into the right first-order hepatic duct (RHD) and contralateral left second-order biliary radicals.

**IIIb:** Infiltration into the left first-order hepatic duct (LHD) and contralateral right second-order biliary radicals.

**Type IV:** Tumor extends bilaterally from the hepatic duct confluence into both second-order biliary radicals of the right and left hepatic ducts, or exhibits multifocal involvement of intrahepatic ducts.

### TABLE S1. MRI sequence parameters

|  | **Parameter** | **T2WI** | **e-THRIVE** | **DWI** |
| --- | --- | --- | --- | --- |
| Avanto 1.5T | Repetition time (TR) (ms) | 168 | 3.95 | 4500 |
|  | Echo time (TE) (ms) | 1.23 | 2.86 | 709 |
|  | Field of view (mm^2^) | 256×192 | 320×187 | 192×153 |
|  | Size of matrix | 256×154 | 320×250 | 192×156 |
|  | Section thickness (mm) | 5 | 3 | 4 |
|  | slice gap (mm) | 1.0 | 0 | 1.1 |
|  | ***b*** value(s/mm^2^) | - | - | 0, 1000 |
| Prisma 3.0T | Repetition time (TR) (ms) | 2391.5 | 3.63 | 5700 |
|  | Echo time (TE) (ms) | 101 | 1.67 | 61 |
|  | Field of view (mm^2^) | 320×320 | 323×258 | 265×226 |
|  | Size of matrix | 320×320 | 323×258 | 135×110 |
|  | Section thickness (mm) | 5 | 2 | 4 |
|  | slice gap (mm) | 1.1 | 0 | 1.1 |
|  | ***b*** value(s/mm^2^) | - | - | 0, 50, 800 |
| Achieva 3.0T | Repetition time (TR) (ms) | 1610 | 3.0 | 1244 |
|  | Echo time (TE) (ms) | 70 | 50 | 1.38 |
|  | Field of view (mm^2^) | 280 × 305 | 280 × 305 | 219×296 |
|  | Size of matrix | 176 × 201 | 100×124 | 208×164 |
|  | Section thickness (mm) | 7 | 7 | 1.46 |
|  | slice gap (mm) | 1 | 1 | 1 |
|  | ***b*** value(s/mm^2^) | - | - | 0, 800 |

|  | contrast agent | contrast agent  (mL/s) | Dose  ( mL/ kg) | Time of four-phase enhanced scan ( s) | | | |
| --- | --- | --- | --- | --- | --- | --- | --- |
|  |  |  |  | Arterial phase | Portal phase | Equilibrium phase | Delay  phase |
| Avanto 1.5T | Gadopentetate dimeglumine | 2 | 0.2 | 20-25 | 60-65 | 115-120 | 180-195 |
| Prisma 3.0T | Gadobenate meglumine | 2 | 0.2 | 25 ±5 | 60 ±5 | 100 ±5 | 180 ±5 |
| Achieva 3.0T | gadobutrol | 2.5 | 0.2 | 20-30 | 60-70 | 120-130 | 180-200 |

### TABLE S2. e-THRIVE contrast-enhanced parameters
